# Beyond boundaries: unraveling innovative approaches to combat bone-metastatic cancers

**DOI:** 10.3389/fendo.2023.1260491

**Published:** 2024-01-08

**Authors:** Huanrong Lan, Bo Wu, Ketao Jin, Yefeng Chen

**Affiliations:** ^1^ Department of Surgical Oncology, Hangzhou Cancer Hospital, Hangzhou, Zhejiang, China; ^2^ Department of Colorectal Surgery, Affiliated Jinhua Hosptial, Zhejiang University School of Medicine, Jinhua, Zhejiang, China; ^3^ Department of Respiratory Medicine, Shaoxing People’s Hospital, Shaoxing, Zhejiang, China

**Keywords:** bone remodeling, metastasis, treatment-related neoplasms, cancers, bone microenvironment

## Abstract

Evidence demonstrated that bones, liver, and lungs are the most common metastasis sites in some human malignancies, especially in prostate and breast cancers. Bone is the third most frequent target for spreading tumor cells among these organs and tissues. Patients with bone-metastatic cancers face a grim prognosis characterized by short median survival time. Current treatments have proven insufficient, as they can only inhibit metastasis or tumor progression within the bone tissues rather than providing a curative solution. Gaining a more profound comprehension of the interplay between tumor cells and the bone microenvironment (BME) is of utmost importance in tackling this issue. This knowledge will pave the way for developing innovative diagnostic and therapeutic approaches. This review summarizes the mechanisms underlying bone metastasis and discusses the clinical aspects of this pathologic condition. Additionally, it highlights emerging therapeutic interventions aimed at enhancing the quality of life for patients affected by bone-metastatic cancers. By synthesizing current research, this review seeks to shed light on the complexities of bone metastasis and offer insights for future advancements in patient care.

## 1 Introduction

Metastatic tumors, unlike primary tumors, present a significant challenge in treatment and have a high mortality rate, resulting in the death of over 90% of patients (1). Systemic therapies, including chemotherapy, targeted therapy, and immunotherapy, have emerged as the primary strategies to combat metastatic cancers, offering improved patient survival rates and enhanced quality of life (2, 3). Metastasis involves a complex process characterized by reduced intercellular cohesion, migration of tumor cells from the primary site through lymph nodes or the bloodstream, and establishment in distant organs while evading the immune system (4). Following the metastasis of tumor cells to secondary locations, angiogenic processes facilitate their growth and survival by providing oxygen and nutrients (5, 6). Breast and prostate cancers have a pronounced inclination for bone metastasis compared to other types of metastatic cancers. Consequently, bones stand as the third most frequently affected site by cancer spread, trailing only behind the lungs and liver (7–9).

The presence of bone metastases gives rise to severe complications, including but not limited to pain, pathological fractures, hypercalcemia, spinal cord compression, and bone marrow aplasia (8, 10). However, existing treatments, including bisphosphonates, denosumab, and radiotherapy, have limitations and can only inhibit bone metastasis without providing a curative solution. As a result, researchers have explored immunotherapeutic approaches like chimeric antigen receptor (CAR) T-cell therapy, depletion of regulatory T-cells (Tregs) using antibodies such as basiliximab and daclizumab, or antibodies targeting cytotoxic T-lymphocyte antigen-4 (CTLA-4) like tremelimumab and ipilimumab, to suppress bone metastasis (11, 12). To uncover innovative therapeutic approaches, it is imperative to deeply comprehend the multifaceted aspects of tumor cell metastasis to the bones and the alterations in the microenvironment after tumor cell migration and homing (13–15).

This review aims to comprehensively summarize bone metastasis’s mechanisms and clinical aspects in cancers. It will discuss the properties and efficacy of established and emerging therapeutic approaches for treating bone-metastatic cancers. By elucidating the current understanding of bone metastasis and therapeutic interventions, this review seeks to contribute to advancing patient care and developing more effective treatment strategies.

## 2 Tumor metastasis mechanisms

Metastasis, a complex and crucial step in cancer progression, is responsible for most cancer-related deaths (16). This process involves a series of intricate events that allow tumor cells to spread from the primary site to distant organs (17). Understanding the underlying mechanisms is vital for developing effective therapeutic strategies. This section delves into the stages of the metastatic process and sheds light on recent findings that elucidate tumor cell dynamics.

### 2.1 Angiogenic mechanisms and cancer stem cells

Within the primary tumor, initiating angiogenic mechanisms ensures the supply of oxygen and nutrients to sustain tumor growth. Moreover, a subset of tumor cells known as cancer stem cells (CSC) can undergo epithelial-mesenchymal transition (EMT), leading to their detachment from the primary tumor mass (18). This process allows CSCs to acquire migratory and invasive properties, enabling them to penetrate the basement membrane and extracellular matrix (ECM) and invade adjacent tissues (19, 20).

### 2.2 Intravasation and extravasation

Upon breaching the basement membrane and ECM, detached tumor cells enter nearby lymphatic vessels or the bloodstream, a process known as intravasation (21). Some circulating tumor cells survive within the circulation, potentially exiting through extravasation into a distant tissue (22). Tumor cell interactions with the basement membrane and ECM are critical in facilitating their entry into the bloodstream and subsequent metastasis (23).

### 2.3 Premetastatic niche formation

Successful metastasis relies on establishing a premetastatic niche, which provides an environment conducive to the attachment and proliferation of disseminated tumor cells (24). This process of niche formation entails dynamic interactions between tumor cells and diverse components of the tumor microenvironment (TME), which includes stromal cells. Additionally, genomic aberrations in end-stage malignancies have been shown to impact metastatic processes (18, 19).

### 2.4 Angiogenic cascades and tumor growth

Upon reaching a secondary site, tumor cells trigger angiogenic cascades to establish a vascular network, facilitating their growth and survival in the new environment. The interaction between tumor cells and stromal cells within the TME, coupled with genomic alterations, plays a pivotal role in determining the capacity of tumor cells to successfully establish and flourish at the secondary site (25, 26).

Recent studies have shed light on the intricate dynamics of tumor cell dissemination and metastasis (27, 28). Understanding these processes in greater detail offers opportunities for developing targeted interventions to disrupt critical steps in the metastatic cascade (29–31). By unraveling the complexities of tumor cell interactions with the microenvironment and genetic factors influencing metastasis, researchers aim to identify novel therapeutic approaches to combat metastatic cancers.

## 3 The most common sites for cancers to metastasize

Although almost all types of human malignancies can spread outside their origin site, some of the most common cancer types, including breast cancer, prostate cancer, lung cancer, kidney cancer, thyroid cancer, colon cancer, pancreatic cancer, bone cancer, and liver cancer have more metastatic properties than other tumors (32). The most common sites for cancer metastasis are the lungs, liver, bones, and brain. Different tissues and organs, such as the lymph nodes, adrenal gland, and skin, could also be the secondary tumor site (**Table 1**). In some cases, the origin of a metastatic tumor is unknown, and this type of cancer is called cancer of unknown primary (CUPS) (43). Prior research indicated that bone metastases were prevalent in approximately 84% of individuals with metastatic prostate cancer. Distant lymph nodes were found to be affected in about 10.6% of cases, while liver metastases were observed in approximately 10.2% of patients. Additionally, thoracic metastases were recorded in roughly 9.1% of the study population (33). Metastatic lung cancer and adenocarcinoma were predominantly found to spread to various organs. The nervous system was affected in approximately 47% of cases, making it the most frequent site of metastasis. Bone metastases were observed in around 39% of patients, while liver involvement was detected in about 35% of cases. Moreover, the respiratory system was affected in approximately 22% of individuals with metastatic lung cancer and adenocarcinoma (34). However, the percentages may vary in men and women for human malignancies, including lung cancer. Another study also reported that bone is the most common site of metastasis for adenocarcinoma and squamous cell carcinoma. In contrast, the most common site for small cell lung cancer (SCLC) metastasis is the liver (44).

**Table 1:** The most frequent sites for cancers to metastasize.

Type of cancer	Sites of metastasis	Ref
Metastatic prostate cancer	• Bone (84%) • Distant lymph nodes (10.6%) • Liver (10.2%) • Thorax (9.1%)	(33)
Metastatic lung cancer and adenocarcinoma	• Nervous system (47%) • Bone (39%) • Liver (35%) • Respiratory system (22%)	(34)
Recurrent endometrial carcinoma	• Pelvic and para-aortic lymph nodes • Peritoneum • Lungs • Vagina • Other atypical sites such as bones, abdominal wall, muscle, intra-abdominal organs, and brain	(35)
Metastatic renal cell carcinoma	• Bone metastasis (10%–49%) • Brain (2%–16%)	(36)
HCC	The most common metastatic site: • Lungs Infrequent extrahepatic metastatic sites: • Abdominal regional lymph nodes • Bones • Diaphragm • Pancreas • Gall bladder • Stomach, colon • Pleura • Peritoneum • Cervical lymph nodes • Shoulder soft tissue • Adrenal gland	(37)
Breast cancer (TNBC, nonbasal, HER2 enriched, basal-like, luminal/HER2, luminal A, and luminal B)	All breast cancer subtypes excluding basal-like tumors: • Bone Compared with luminal A tumors, luminal/HER2 and HER2-enriched tumors were associated with: • Brain • Liver • Lung Basal-like tumors: • Brain • Lung • distant nodal • A remarkably lower rate of metastasis to bone and liver TNCB nonbasal tumors disclosed similar metastatic sites; nonetheless, they were not accompanied by fewer liver metastases	(38)
Colorectal cancer	43% of patients with right colon cancer, 54% with left colon cancer, and 52% of patients with rectal cancer: • Liver 33% of patients with right colon cancer: • Peritoneal metastases 28% of patients with rectal cancer: • Lung	(39) (40)
Head and neck squamous cell carcinoma	• Pulmonary metastases (66%) • Bone (22%) • Liver (10%) Other sites: • Mediastinum • BM • Skin	(41)
Metastatic urothelial carcinoma of the urinary bladder	• Lymph node (25%) • Bone (24%) • Urinary (23%) • Lung (23%) • Liver (3%)	(42)

Results obtained through various imaging modalities revealed that in cases of recurrent endometrial carcinoma, the most prevalent sites of metastasis were the pelvic and para-aortic lymph nodes, peritoneum, lungs, and vagina. However, it’s worth noting that atypical sites like bones, abdominal wall, muscle, intra-abdominal organs, and even the brain could also serve as secondary sites where metastatic tumor cells were found to migrate and establish themselves (35). In metastatic renal cell carcinoma, the rates of bone metastasis were (10%–49%), and metastasis to the brain was (2%–16%) (36). In a comprehensive examination of hepatocellular carcinoma (HCC) autopsy cases, the presence of extrahepatic metastases was identified in 68% of the patients. The lung emerged as the most prevalent site of metastasis. Additionally, several infrequent extrahepatic metastatic sites were also observed, which included the abdominal regional lymph nodes, bones, diaphragm, pancreas, gall bladder, stomach, colon, pleura, peritoneum, cervical lymph nodes, shoulder soft tissue, and adrenal gland (37). An investigation evaluated the common metastatic sites in different subtypes of breast cancer, including triple negative (TNBC) nonbasal, HER2 enriched, basal-like, luminal/human epidermal growth factor receptor 2 (HER2), luminal A, and luminal B. Outcomes showed that bone was the most frequent metastatic site in all breast cancer subtypes excluding basal-like tumors. Moreover, luminal/HER2 and HER2-enriched tumors were associated with a pointedly higher rate of metastasis to the brain, liver, and lung compared with luminal A tumors. Basal-like tumors were accompanied by a higher rate of metastasis to the brain, lung, and distant nodal but a remarkably lower rate of metastasis to bone and liver. Furthermore, TNBC nonbasal tumors disclosed similar metastatic sites; nonetheless, they were not accompanied by fewer liver metastases (38). Recently, a study on colorectal cancer (CRC) demonstrated that metastatic sites differed meaningfully from primary tumor sites. Metastasis to the liver occurred in 43% of patients with right colon cancer, 54% in left colon cancer, and 52% in patients with rectal cancer. Moreover, in 33% of patients with right colon cancer, peritoneal metastases were most common, while lung metastases were common in 28% of patients with rectal cancer (40). Therefore, the primary tumor site could affect the survival rate of patients with metastatic CRC. Patients with bone metastasis have the most significant prognosis in stage IV breast cancer, whereas patients with brain metastasis are the most aggressive subclass (39). Head and neck squamous cell carcinoma exhibit distant metastases in 66% of cases, with the lungs being the most common site of metastasis. Nonetheless, other organs can also serve as metastatic locations in this type of cancer. Approximately 22% of distant metastases occur in the bone, 10% in the liver, and additional sites include the mediastinum, bone marrow (BM), and skin (41). An investigation involving 7543 patients diagnosed with metastatic urothelial carcinoma of the urinary bladder revealed the distribution of metastatic sites. The study indicated that the most common locations of metastases were the lymph nodes, accounting for approximately 25% of cases. The bone and urinary systems followed closely, with both sites having a prevalence of around 24% and 23%, respectively. Similarly, lung metastases were found in approximately 23% of patients, while liver metastases were relatively less frequent, occurring in approximately 3% of the study population (42).

## 4 Bone remodeling and involved key factors

According to the available knowledge, hematopoietic stem and precursor cells (HSPCs)-derived osteoclasts can create irregular resorption grooves and an acidic condition in the bone to eliminate calcium and digest non-collagenous and collagenous proteins from the bone ECM. Aside from calcium, growth factors are also released during this resorption process (45). By contrast, osteoblasts derived from mesenchymal stem cells (MSCs) deposit matrix mineralized to repair these resorption regions. Embedded osteocytes in the mineral of the bone play a role as mechanosensors and communicate via gap junctions and dendrites to regulate osteoclasts’ and osteoblasts’ actions. In addition to producing bone remodeling cytokines, osteocytes produce other essential mediators. Osteocytes, osteoblasts, activated T cells, and tumor cells all release receptor activator of NF-κB ligand (RANKL), a necessary mediator for osteoclastogenesis and osteoclast survival and function.

**Figure 1:**
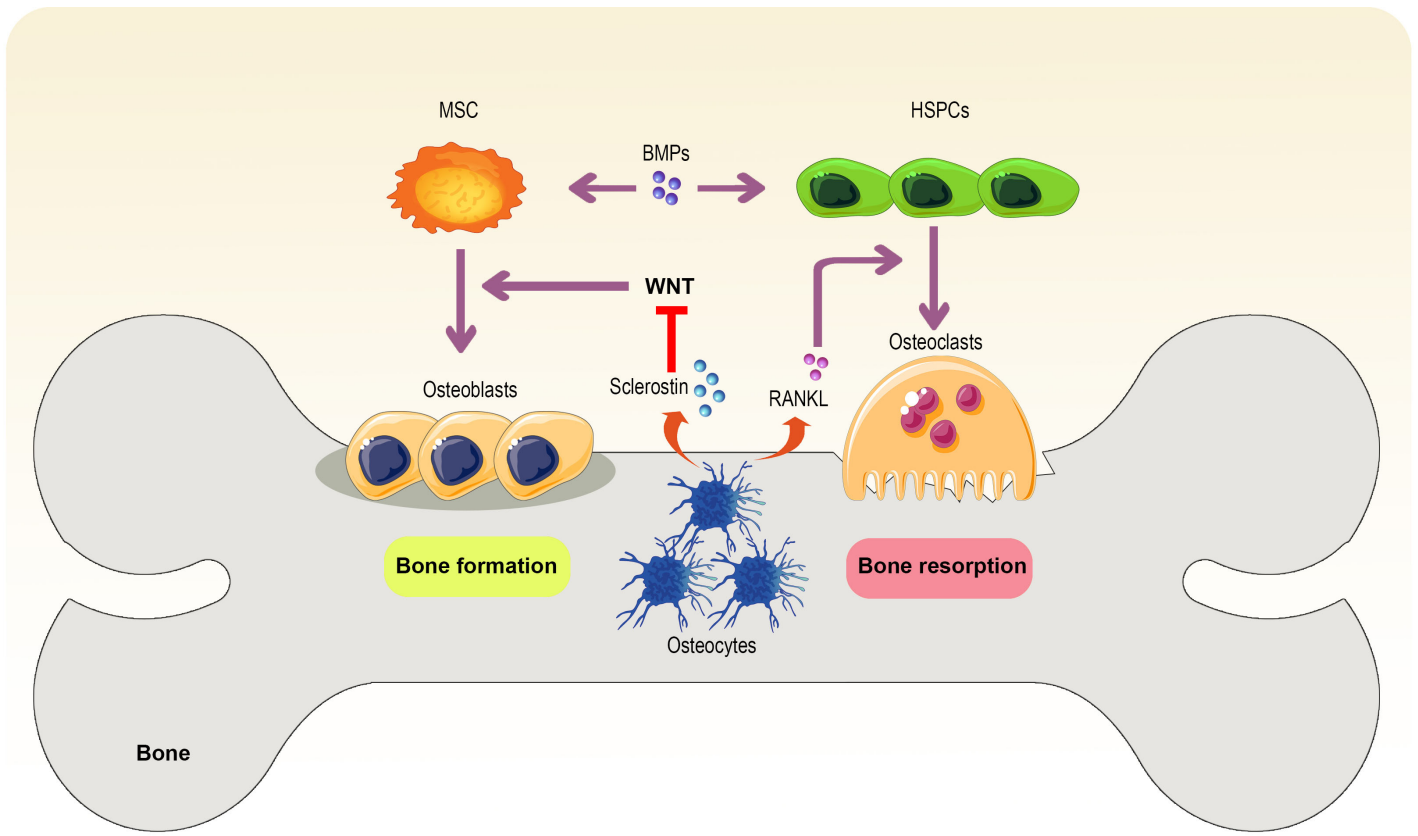
Bone remodeling. The interaction between osteoblasts and osteoclasts under the influence of bone environment conditions and mediators secreted by osteoblasts and other cells ultimately leads to bone formation and resorption. In the meantime, MSCs help bone formation, but HSPCs support osteoclasts and bone resorption, in which osteocyte-derived RANKL and sclerostin also play an essential role in promoting this phenomenon and bone formation suppressing.

Furthermore, Dickkopf-related proteins 1 (DKK1) and sclerostin are released by osteocytes, inhibiting osteoblastogenesis from MSCs by suppressing WNT signaling. As well as communicating through bone morphogenetic proteins (BMPs), MSCs and HSPCs are also involved in dynamic bone remodeling and reciprocally maintaining bone homeostasis (**Figure 1**). The canonical WNT signaling pathway induces precursors into osteoblast differentiation. Furthermore, this pathway exerts an inhibitory effect on bone resorption through the upregulation of osteoprotegerin (OPG) and the downregulation of RANKL expression in osteoblasts (46).

RANKL is a crucial tumor necrosis factor-related mediator, and various tissues and organs, including bone, brain, spleen, lungs, lymph nodes, heart, mammary gland, thymus, skin, kidneys, and skeletal muscle, can express it (47, 48). Moreover, RANKL is expressed amply by osteoblasts, chondrocytes, immune, stromal, mesenchymal, and spleen cells (49). However, mature osteoblasts are considered the main RANKL source regulating BM macrophage-derived osteoclastogenesis (50, 51). Parathyroid is the main endocrine gland responsible for secreting PTHrP. It should be noted that PTHrP is occasionally secreted by some malignant cells (52). An early discovery was that PTHrP is a peptide hormone contributing to humoral hypercalcemia during cancerous conditions (53). While bone development is underway, PTHrP determines structural mineralization and chondrogenesis (54). In the early studies, PTHrP was thought to promote bone demineralization in malignant patients due to its high osteoclast activity. However, later investigations proved its osteogenic potential due to amplified bone mass in animals and human undertreatment with PTHrP (54–57). However, another study reported that due to suppressing both Runx2 and Runx3 transcription factors, PTHrP inhibited chondrocyte proliferation in primary chondrocytes isolated from wild mice (58).

## 5 Bone metastasis

As discussed in the previous section, bone is one of the most common sites of metastasis, usually associated with poor prognosis and short-term survival rates for cancer patients (59). Interestingly, bone metastases are more reported than primary bone cancers, especially in adults. In this section, the characteristics of bone metastasis are discussed.

### 5.1 Bone-metastatic cancers

Evidence revealed that 65-75% of breast cancers are associated with bone metastasis. Breast cancer and bone metastasis patients are associated with poor prognosis and low survival rates, 2–3 years upon diagnosis (60). Severe pain, spinal cord compression, bone marrow aplasia, hypercalcemia, reduced mobility, osteolysis, and bone fractures are the leading causes of increased morbidity in breast cancer patients with bone metastasis. After the release of inflammatory mediators by tumor cells and BME components, the alteration of the BME and bone homeostasis leads to mechanical pressure and bone pain (10). The axial skeleton connected to BM content and hematopoiesis is the most common target of metastasis in breast cancer. In patients with advanced prostate cancer, bone metastases are common, leading to bone pain, fractures, and increased mortality. The bone tissue provides a supportive microenvironment for the growth and progression of tumor cells. It has been discovered that interactions between invasive tumor cells, bone-forming osteoblasts, and bone-resorbing osteoclasts play a crucial role in the development of prostate cancer manifestations.

For example, parathyroid hormone-related peptide (PTHrP) can trigger the upregulation of RANKL expression and release various growth factors in the BME. This, in turn, activates bone-resorbing osteoclasts, leading to bone resorption. These complex interactions contribute to the progression and impact of prostate cancer within the bone tissue (61). Thyroid cancer patients experience reduced survival when metastasis occurs in distant organs. Bone metastasis, particularly in follicular thyroid cancer, is prevalent and often leads to symptoms such as pain, bone fractures, and spinal cord compression, significantly impacting patients’ quality of life. Furthermore, bone metastases in follicular thyroid cancer are typically linked to elevated bone turnover markers, reflecting the increased activity in the bone microenvironment caused by the cancer cells (62).

### 5.2 Types of bone metastases

Concerning the principal process by which normal bone tissue undergoes remodeling due to the presence of bone metastases, they can be classified into three main types: osteoblastic, osteolytic, and mixed, each presenting distinct interference mechanisms. The osteoblastic type commonly detects carcinoid, prostate cancer, Hodgkin lymphoma, small cell lung cancer, and medulloblastoma, which are recognized by the deposition of new bone formation. It has been shown that, in some cases, the formation of new bone tissue does not always occur after bone resorption (8). As a result, osteoblasts’ activation is crucial in forming these new bone tissues. Factors such as bone morphogenic proteins (BMP), transforming growth factor (TGF), endothelin-1, and core-binding factor alpha 1 (Cbfa1) are involved in the proliferation, activation, and differentiation of osteoblasts (63, 64).

Moreover, PTHrP could be cleavaged by prostate-specific antigen (PSA), leading to a decrease in osteoclast bone resorption and a disturbance of the balance between osteoblasts and osteoclasts (65). Osteolytic is another type of bone metastasis in which osteoclasts are activated, destroying bone tissue (66, 67). Osteolytic bone metastasis presents in breast, thyroid, melanoma, renal cell carcinoma, multiple myeloma, non-Hodgkin lymphoma, non-small cell lung cancer, and Langerhans-cell histiocytosis (10). However, ischemia following the compression of vasculature in the late stages of cancer could be another cause of osteolytic lesion development (8). It has been theorized that increased PTHrP by tumor cells in the BME and upregulation of RANKL play a pivotal role in forming osteoclasts and other osteolytic lesions (68). Finally, a patient could have both osteoblastic and osteolytic lesions (mixed type) observed in breast, squamous, and gastrointestinal cancers (69).

### 5.3 Mechanisms of bone metastasis

**Figure 2:**
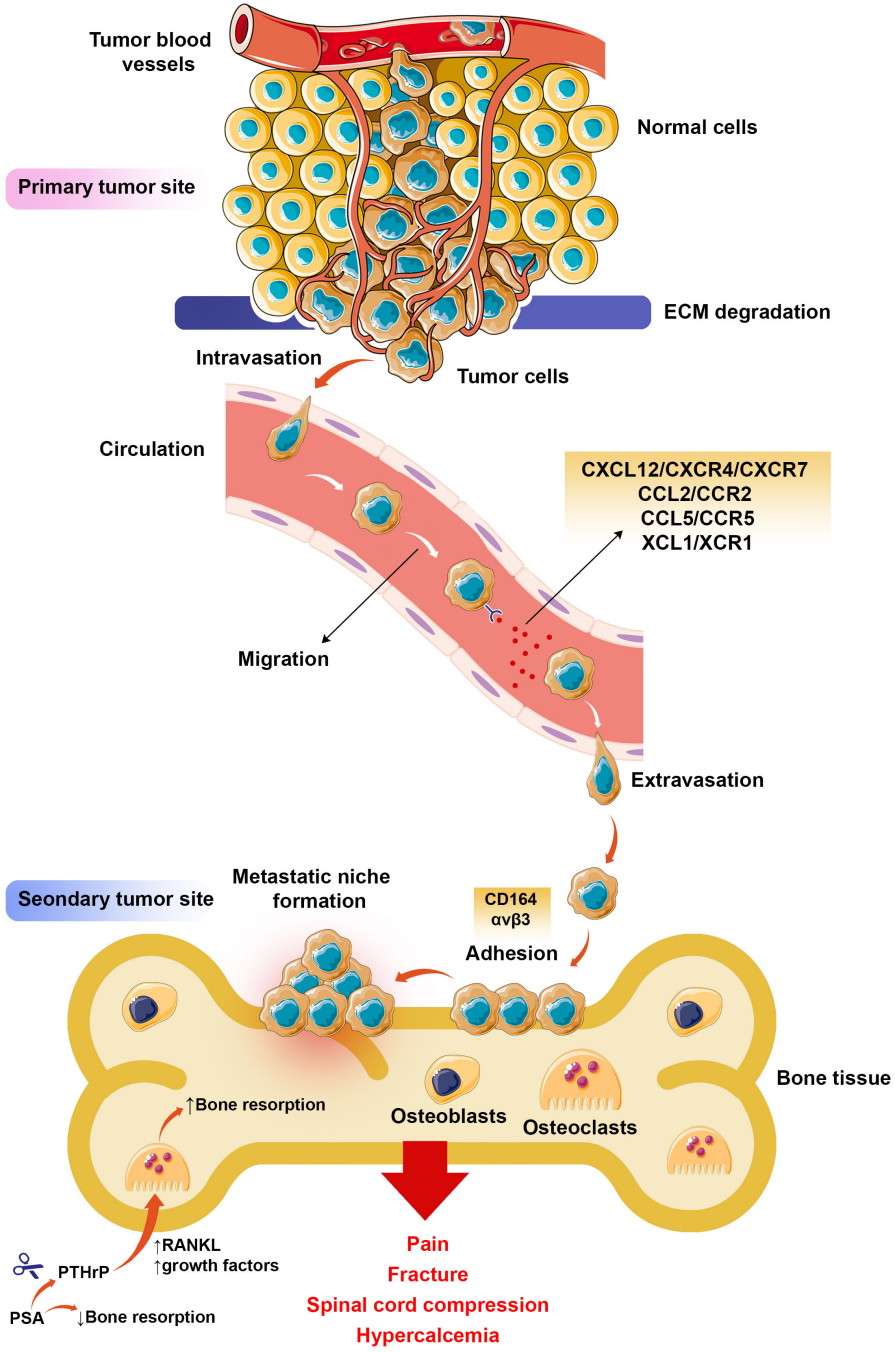
Bone metastasis process. After activating a group of tumor cells in the primary tumor tissue, these cells enter the bloodstream by degradation of ECM (intravasation) and begin migrating to distant organs with chemokine axes such as CXCL12/CXCR4. Upon reaching the target organ, such as bone, exit from the arteries (extravasation), and after expressing the adhesion molecules and attaching to the bone tissue, they form the premetastatic niche, where the tumor cells grow and develop, resulting in pain, fracture, spinal cord compression, and hypercalcemia in patients with bone-metastatic malignancies.

Bone metastasis is a highly organized and controlled process, as depicted in **Figure 2**. It involves a complex interplay between the tumor and bone, disrupting the bone matrix and tumor progression (70). The initial step in bone metastasis entails the escape and spread of tumor cells from the primary site, achieved by breaking down ECM proteins. This process is crucial for tumor cells to enter the circulation and migrate to secondary sites, and matrix metalloproteinases (MMPs) play a pivotal role in ECM protein degradation (71). Increased levels of MMPs have been observed in various human malignancies, indicating a poor prognosis. Moreover, the MMP family is thought to participate in angiogenic mechanisms (72). Once detached from the primary tumor mass, the adhesion and invasion of tumor cells are facilitated by chemokines and adhesion molecules, such as CD164 and αvβ3 integrins (73).

Furthermore, cyclooxygenase-2 (COX-2) expression can induce the adhesion and proliferation of cancer cells (74). CXCL12 plays a significant role in bone metastasis among chemokines and is often expressed in common metastatic sites such as BM (75). The receptors known for CXCL12 are CXCR4 and CXCR7 and are expressed by tumor cells and a range of other cells, such as immune cells, fibroblasts, and endothelial cells (76). In addition to cell mobilization from BM, the CXCL12/CXCR4/CXCR7 axes can participate in the growth and development of tumor cells and angiogenesis (77). However, other chemokine axes, such as CCL5/CCR5 and XCL1/XCR1, promote the proliferation and migration of tumor cells (78, 79). Metastasis formation heavily relies on the bone niche, which plays a fundamental role by expressing elevated levels of CCL2, an additional chemoattractant factor. This heightened expression of CCL2 attracts and recruits tumor cells, thereby contributing significantly to the process of tumorigenesis (75).

Other tumor cell-derived proteinases, such as uPA82 and ADAM, have been reported to participate in bone matrix degradation and invasion of tumor cells into bones (80, 81). Maintaining tumor cell proliferation following metastasis is essential for tumor survival and development. In bone metastasis, bone resorption, and bone formation imbalance disrupt the physiologic bone remodeling (82). A variety of growth factors secreted by osteoblasts following osteoclastic bone resorption in the BME are also involved in tumor cell growth and survival (82). Recent research has highlighted the crucial role of osteoclasts in osteolytic bone metastasis, as they are responsible for digesting the bone matrix and indirectly facilitating tumor colonization. An investigation has shown that the IL-20R subunit β (IL-20RB) significantly promotes a direct tumoral response to osteoclasts. In the context of bone metastasis in lung cancer, the expression of IL-20RB is associated with the growth and progression of lung cancer cells within the bone. During this process, tumor cells stimulate osteoclasts to release IL-19, which acts as the ligand for IL-20RB. Consequently, IL-19 promotes the activation of IL-20RB-expressing tumor cells, activating the JAK1/signal transducer and activating the transcription 3 (STAT3) signaling pathway. This signaling cascade further enhances tumor cell proliferation within the bone microenvironment. Therefore, designing neutralizing antibodies or antagonists to block IL-20RB can be a potential therapeutic tactic to inhibit bone metastasis (83).

Sclerostin, a protein produced by bone cells called osteocytes, hinders the activity of osteoblasts, which are responsible for bone formation, by obstructing the canonical Wnt signaling pathway (84). Inhibiting sclerostin, whether through genetic means or medications, has been proven to boost bone formation and is sanctioned for the treatment of osteoporosis (85, 86). Recently, a study explored the role of sclerostin in bone formation and its potential impact on the spread of breast cancer to the bone (87). During the research, it was discovered that a particular type of breast cancer cell, namely MDA-MB-231, exhibited a robust response to Wnt3a, a Wnt protein responsible for activating the canonical Wnt signaling pathway. Remarkably, when these MDA-MB-231 cells were treated with an anti-sclerostin antibody, there was a notable increase in bone metastasis formation, while other breast cancer cell lines did not show a significant effect in response to the treatment. Of particular interest, the administration of the anti-sclerostin antibody resulted in the accumulation of β-catenin, a protein that plays a key role in the Wnt signaling pathway, specifically within the MDA-MB-231 cells located in the bone microenvironment. This suggests a potential link between the activation of canonical Wnt signaling through the accumulation of β-catenin and the increased propensity for bone metastasis formation in MDA-MB-231 breast cancer cells. In addition to its role in activating the canonical Wnt signaling pathway, Wnt3a was found to promote the formation of tumorspheres, which are clusters of cancer cells exhibiting stem cell-like characteristics, in MDA-MB-231 cells. However, Wnt3a did not appear to have a significant impact on the individual proliferation and migration of these cells.

Furthermore, the research showed that treating MDA-MB-231 cells with the anti-sclerostin antibody resulted in a substantial increase in the number of osteoclasts, which are cells responsible for bone breakdown, as well as their precursor cells in the bone metastatic sites (87). This suggests that blocking sclerostin amplifies canonical Wnt signaling in breast cancer cells that are responsive to Wnt ligands, consequently promoting increased bone metastasis. This effect might occur, at least in part, by stimulating stem cell-like properties in cancer cells and facilitating osteoclastogenesis in the bone microenvironment.

Taken together, these findings shed light on the intricate mechanisms through which blocking sclerostin can influence Wnt signaling, tumor sphere formation, and osteoclastogenesis in the context of bone metastasis, particularly in breast cancer cells that exhibit a strong response to Wnt ligands like Wnt3a.

A study was conducted to investigate the role of TEX41 in bone metastasis of lung adenocarcinoma (LUAD) (88). The researchers utilized various methods, including bioinformatics analysis, quantitative PCR, fluorescence *in-situ* hybridization (FISH), and *in vivo* experiments with nude mice. They focused on investigating the functions and molecular mechanisms of TEX41, its association with Runx2, and its impact on various aspects of LUAD (lung adenocarcinoma) cell behavior, including proliferation, migration, invasion, and metastasis. The results showed that TEX41 expression was notably higher in LUAD bone metastasis (BM) tissue, suggesting a poorer prognosis for LUAD patients with bone metastasis. Knocking down TEX41 reduced LUAD cell migration and metastasis, whereas overexpressing TEX41 promoted these processes. X-ray and histological staining confirmed that TEX41 supported bone metastasis in LUAD.

The study also revealed that TEX41 induced autophagy in LUAD cells, as evidenced by changes in autophagy-related markers. Further investigation through FISH analysis demonstrated that TEX41 and Runx2 were colocalized in the nucleus, and TEX41 was found to regulate the expression of Runx2. Inhibiting Runx2 counteracted the effects of TEX41 on LUAD cell migration, invasion, metastasis, and autophagy. Furthermore, the study discovered that the role of TEX41 in metastasis partially relied on autophagy, and the phosphoinositide 3-kinase (PI3K)-AKT pathway played a significant role in TEX41-mediated autophagy. The study provides valuable insights into the molecular mechanisms underlying the involvement of TEX41 in LUAD bone metastasis. TEX41 was found to promote autophagy in LUAD cells by upregulating Runx2, and this process mediated LUAD cell migration, invasion, and bone metastasis. These findings contribute to a better understanding of the role of TEX41 in LUAD metastasis to the bone and offer potential targets for future therapeutic interventions (88).

A different study delved into the constraints of immune checkpoint therapy for prostate cancer, attributing them to the unique molecular attributes of prostate cancer cells and the suppressive environment within the bone TME (89). The researchers aimed to identify subgroups of prostate cancer patients suitable for immune checkpoint therapy. They investigated the role of a specific protein called BHLHE22 in prostate cancer bone metastasis and immunosuppressive bone TME. The findings of the study revealed that in bone metastatic prostate cancer, there is an increased expression of a gene called BHLHE22, which contributes to the establishment of an immunosuppressive bone tumor microenvironment (TME). BHLHE22 was found to be responsible for the elevated levels of CSF2, which, in turn, promoted the infiltration of immunosuppressive neutrophils and monocytes, ultimately leading to a prolonged state of compromised T-cell function. The researchers uncovered that BHLHE22 achieves this effect by binding to the CSF2 promoter and recruiting PRMT5, forming a transcriptional complex that epigenetically activates CSF2 expression (89). To address the resistance of BHLHE22-positive tumors to immune checkpoint therapy, the investigators explored a combined treatment approach that targeted both protein arginine methyltransferase 5 (PRMT5) and colony-stimulating factor 2 (CSF2). This strategy aimed to neutralize the immunosuppressive impact of neutrophils and monocytes within the tumor microenvironment.

In a tumor-bearing mouse model, inhibition of Csf2 and Prmt5 improved immune checkpoint therapy efficacy in BHLHE22-positive tumors. Together, the study provides insights into the immunosuppressive mechanism driven by BHLHE22 in prostate cancer and proposes a potential combination therapy approach involving immune checkpoint therapy and targeting CSF2 and PRMT5 for patients with BHLHE22-positive Prostate cancer with bone metastasis (89).

Bone metastasis in lung cancer is characterized by abnormal differentiation and dysfunction of osteoclasts (90). A study highlights the role of exosomes derived from lung cancer cells in promoting osteoclast differentiation and bone metastasis. The findings suggest that exosomal HOTAIR may contribute to the abnormal bone remodeling seen in lung cancer bone metastasis through its effects on the TGF-β/PTHrP/RANKL pathway (91). The investigation revealed a notable increase in the expression of a long non-coding RNA named HOTAIR in exosomes derived from lung cancer cell lines A549 and H1299 compared to those obtained from normal lung fibrocytes. Additionally, it was observed that when HOTAIR was overexpressed in the exosomes of A549 and H1299 cells, it actively stimulated osteoclast differentiation.

Additionally, the researchers found that these lung cancer-derived exosomes (A549-Exos and H1299-Exos) targeted bone tissues and significantly inhibited bone formation *in vivo*. Mechanistically, it was discovered that exosomal HOTAIR played a role in promoting bone resorption by targeting the TGF-β/PTHrP/RANKL pathway. The specific molecular details of this interaction were not provided in the passage, but it suggests that exosomal HOTAIR may influence the signaling pathway involved in bone resorption (91). This study highlights the role of exosomes derived from lung cancer cells in promoting osteoclast differentiation and bone metastasis. The findings suggest that exosomal HOTAIR may contribute to the abnormal bone remodeling seen in lung cancer bone metastasis through its effects on the TGF-β/PTHrP/RANKL pathway.

A recent study aimed to identify microRNAs (miRNAs) linked to bone metastasis in Gleason Score (GS) 3 + 4 prostate cancer (92). The research identified three miRNAs, miR-1-3p, miR-143–3p, and miR-145–5p, associated with bone metastasis in GS 3 + 4 prostate cancer. In laboratory experiments, these miRNAs were found to promote the proliferation and migration of prostate cancer cells. Further investigation revealed that the target gene LASP1 was a common target of these three miRNAs, and this was confirmed through a luciferase assay. Immunohistochemistry analysis indicated that elevated LASP1 levels correlated with higher Gleason Scores, advanced pathological stages, and metastasis. Additional experiments demonstrated that suppressing LASP1 with siRNA significantly hindered the proliferation and migration of prostate cancer cells, while overexpressing LASP1 had the opposite effect, promoting these processes. Bioinformatics analysis suggested that LASP1 functioned through the Wnt signaling pathway, and it was found that LASP1 interacted with β-catenin to activate this pathway. The study revealed that miR-1-3p, miR-143–3p, and miR-145–5p were associated with bone metastasis in GS 3 + 4 prostate cancer. LASP1 served as a common target of these miRNAs and was implicated in activating the Wnt signaling pathway through its interaction with β-catenin (92). These findings provide valuable insights into the molecular mechanisms driving prostate cancer progression and suggest potential therapeutic targets for managing bone metastasis in GS 3 + 4 prostate cancer.

### 5.4 Clinical manifestations

Bone metastasis is usually reported in breast cancer for up to 20 months. However, this time can be reduced to six months in other bone-metastatic cancers, such as non-small cell lung cancer (93, 94). In patients with prostate cancer with bone metastasis, in which good performance status, the axial skeleton is involved, and after treatment with androgen inhibitors, survival increases to 53 months. While in patients with poor performance status and the presence of visceral disease, survival is reduced to 30 months (95). As discussed, bone metastases are the leading cause of morbidity, characterized by weakened mobility, severe pain, spinal cord compression, pathologic bone fractures, hypercalcemia, and BM aplasia (7).

#### 5.4.1 Bone pains

Almost all the patients suffering from unlocalized bone-metastatic cancers complain of bone pain. These pains worsen at night and could have mechanical or inflammatory origins (96). Tumor cells in the BME release inflammatory mediators, inducing periosteal irritation, intraosseous nerves, and inflammatory-based pains. On the other hand, mechanical pains are associated with the mass tumor effect or its pressure within the bone tissue, which weakens the bone and causes pain due to activity and pressure on the bone tissue (66). It has been revealed that suppressing osteoclastic bone reabsorption can decrease bone pain (96). Back pain progress in 20-30% of patients with breast cancer and 15% of patients with lung cancer may be due to spinal cord compression that could be confirmed by an abnormal spinal radiograph (66). Radiofrequency (RF) ablation has been reported to reduce the pain score in bone metastases (97).

#### 5.4.2 Hypercalcemia

A frequent metabolic complication in bone metastases is hypercalcemia. Focal and generalized tumor cells-mediated osteolysis, dysregulated calcium reabsorption by renal tubular, and decreased renal glomerular function are the most important reasons for hypercalcemia. However, increased PTHrP levels by breast cancer cells, deposition of Bence-Jones proteins and impaired renal function in multiple myeloma, and hyperproduction of active vitamin-D metabolites in some lymphomas lead to bone resorption and intestinal absorption of calcium (15, 98–100). Untreated hypercalcemia can lead to gastrointestinal, cardiac, central nervous systems (CNS), and kidney complications. Furthermore, hypercalcemia inhibits the release of parathyroid hormone, increasing PTHrP and osteoclast-mediated bone resorption (8).

#### 5.4.3 Pathologic bone fractures

In 10-30 percent of patients with bone-metastatic cancers, pathologic bone fractures occurred regularly in proximal parts of the long bones and femur (50% of cases). Moreover, rib fractures also have been reported to lead to vertebral collapses, kyphoscoliosis, and lung disorders (66, 101). Tumor epidural extension into the spine and long bone fractures play a significant role in causing disability due to bone metastasis and pathological fractures. Bone pain may also be an influential predictive factor in the possibility of emerging a pathological fracture (8, 102).

### 5.5 Diagnosis

Upon confirming one of the mentioned clinical manifestations, a complete blood count (CBC), measuring serum levels of calcium, phosphorus, 25-hydroxyvitamin D, parathyroid hormone, alkaline phosphatase (ALP), creatinine, thyroid-stimulating hormone (TSH), as well as protein electrophoresis and imaging can be used to screen for bone metastases (103). For bone pain evaluation, plain radiography is essential; however, this technique has low sensitivity despite its high specificity because X-rays cannot detect metastatic lesions in the early stages of cancer (104). Bone scintigraphy is another susceptible method with low specificity that provides data about the activity of osteoblasts, skeletal vascularity, and bone metabolic reaction to traumatic, neoplastic, or inflammatory disorders (103). Magnetic resonance imaging (MRI) is usually employed to detect spinal cord compression and assess the extent of BM involvement by the tumor (105).

Moreover, the computerized tomography (CT) scan could diagnose bone destruction and sclerotic deposits and detect localized lesions for biopsy (103, 106). The evaluation of metabolic activity can directly measure the presence of a tumor. Positron emission tomography (PET) is a high-sensitive imaging technique that can evaluate metabolic activity (107). The advantages of PET include identifying bone resorption sites that cannot be detected by other diagnostic methods and detecting metastases to non-bone tissues (108). However, it has been revealed that conventional imaging methods such as radiographs, bone scintigraphy, MRI, and CT are nonspecific and insensitive for treatment response monitoring in a clinically relevant time frame. In this context, other techniques such as molecular and hybrid imaging systems, including whole-body MRI, PET/CT, and single-photon emission computed tomography (SPECT)/CT with diffusion-weighted imaging are more accurate diagnostic tools for skeleton staging by quantifying the association between the BME and tumor cells biologic processes than conventional imaging methods, permitting earlier personalized therapy (109). The radiomics nomogram is another technique that combines the multi-parametric MRI-based radiomics signature and clinical risk factors, promoting personalized estimation of bone metastases in newly diagnosed patients with prostate cancer (110). Recently, it has been reported that personalized finite element (FE) computer models can predict the risk of fracture in femoral bone metastases more than clinical assessments according to the involvement of axial cortical on conventional radiographs in advanced cancer patients (111).

## 6 Available and novel therapies

Because they are not curative, treatment of bone metastases usually aims to prevent metastasis to the bone tissue and tumor progression. Combining surgery, radiotherapy, chemotherapy, and immunotherapy can help prevent tumor progression. In this section, due to numerous studies that have discussed traditional therapies, these methods have been briefly explained, and the focus of this study is more on novel therapeutic tactics and the possibility of using them for the treatment of bone-metastatic cancers.

### 6.1 Conventional therapies

It has been shown that in breast and renal cancers, the extent of metastatic lesions carries the risk of fracture, which is also a common complication of bone metastases, resulting in pain and disability (101, 112). Surgery is a standard method to remove metastatic lesions from the long bones and pelvis/acetabulum. However, surgical techniques, such as total en bloc spondylectomy (TES), are invasive and can cause blood loss (113). The prosthetic implant insertion and plate osteosynthesis are performed surgically to prevent possible fractures (114). To increase the effectiveness of surgery, embolization and radiation therapy are also used to treat bone-metastatic cancers (115–117). For instance, external beam radiation therapy (EBRT), a pain relief treatment for patients with bone-metastatic cancers, can synergistically prevent possible fractures with surgery (118). However, some advantages of surgical, perioperative morbidity, and mortality in patients with bone-metastatic cancers should be considered (119). Another research provided evidence that linear accelerator-based radiosurgery for bone oligometastases originating from prostate cancer resulted in minimal toxicity events, a high rate of local control, and extended periods without the need for subsequent systemic treatments after the use of single-fraction stereotactic radiosurgery (SRS) in patients with oligo recurrent prostate cancer. These positive outcomes highlight the importance of considering the option of rescheduling systemic treatments in individuals diagnosed with oligometastatic prostate cancer who undergo SRS (120).

Hormonal therapies are considered the first treatment option in cancer patients who are hormone responders. For example, in patients with estrogen receptor-expressing breast cancer, selective estrogen receptor modulators (SERMs) such as tamoxifen are used as a treatment (121, 122). Correspondingly, in patients with metastatic prostate cancer, to achieve inhibition of metastatic mechanisms and reduce PSA levels, androgen deprivation (ADT) treatment is performed by employing orchiectomy, anti-androgens, and gonadotropin-releasing hormone antagonists or agonists (123). However, the clinical outcome of hormonal therapy in patients with bone metastatic prostate cancer depends on several factors such as bone scan index (BSI), hot spot number (HSN), and race of patients (124). Among patients with bone metastatic prostate cancer, the BSI and HSN significantly influence the 3-year mortality (124).

Another long-used treatment option for bone-metastatic cancers is utilizing high-affinity radioisotopes for bones with different physical properties, such as phosphorus-32. This radioisotope is commonly used to treat metastatic prostate and breast cancers. Common β-emitting radioisotopes for treating bone metastases are samarium-135 (135Sm) and strontium-89 (89Sr). Radioisotopes emit α- or β-particles and deliver damaging radiation to tumor cells (123, 125).

Bisphosphonates are another standard treatment for bone-metastatic cancers because they have a high affinity for the surface of bones that undertake bone resorption. These drugs are classified based on having a specific group, such as nitrogen, and are involved in promoting the apoptosis of osteoclasts by inhibiting protein isoprenylation or disrupting mitochondria (126). Moreover, some of these drugs, including clodronate and pamidronate, inhibit angiogenesis by inhibiting vascular endothelial growth factor (VEGF) and hypoxia-inducible factor 1-alpha (HIF-1α), and others inhibit the adhesion, invasion, and migration of tumor cells (127, 128). However, bisphosphonates such as zoledronic acid could be associated with adverse effects such as jaw osteonecrosis in patients with bone-metastatic cancers (129).

Due to the involvement of different pathways in the pathogenesis of bone metastases, inhibition of these axes, the most important of which are the RANK/RANKL, CXCL12/CXCR4, TGF-β, HIF-1, Wnt/Ras, and PI3K signaling pathways, has also been considered by researchers in the last decade, and several of these inhibitors are currently under clinical evaluation (130–134).

Local surgery, radiation, and systemic tactics such as chemotherapy and targeted therapy are currently the backbones of metastasis inhibition. These treatments are often effective in reducing metastatic tumor mass; however, they do not specifically target the metastatic phase or the regenerative progenitors that remain after the therapeutic removal of macrometastases (135).

### 6.2 Novel therapeutic approaches

Existing therapies, such as inhibitors of related pathways, are ineffective in treating metastatic bone cancers. For instance, monitoring patients with metastatic breast cancer under treatment with Denosumab (anti-RANKL fully human IgG2 monoclonal antibody) demonstrated that the expression of RNAKL on circulating tumor cells (CTCs) is a pivotal step in the metastatic process affects the effectiveness of Denosumab (136). The efficacy of denosumab and zoledronic acid in treating bone metastases in patients with solid tumors and multiple myeloma was evaluated in a meta-analysis. Based on the analysis of four separate randomized controlled trials by this study, patients in the denosumab group had a remarkable delay in the occurrence of skeletal-related events for both the first and subsequent incidences. Denosumab was found to be linked to a higher occurrence of hypocalcemia and osteonecrosis of the jaw compared to zoledronic acid. However, it was also associated with a lower incidence of renal toxicity and acute phase reactions compared to zoledronic acid. Despite the potential risk of jaw osteonecrosis and hypocalcemia, the data suggest that denosumab holds promise as a treatment for multiple myeloma and solid tumor bone metastases. Moreover, measures for preventing and managing these adverse effects have been identified, which can help mitigate their impact (137). Additionally, in postmenopausal women with breast cancer, research findings indicated that adjuvant denosumab led to a reduction in the occurrence of fractures associated with aromatase inhibitor treatment in early breast cancer. However, despite this positive outcome, the large randomized D-CARE study (NCT01077154) did not achieve its primary objective of improving bone metastases-free survival in this group of patients (138).

On the other hand, monotherapy with chemotherapeutic drugs is limited due to improper distribution and increased expression of some molecules in the BME, such as RANKL, inducing a vicious cycle in bone metastasis. For this reason, researchers are looking for a way to increase the effectiveness of existing therapies through combination therapy (**Table 2**). For instance, combining Denosumab with nano-encapsulated docetaxel (an anticancer drug) was more effective than alone in prostate cancer. Using nano-encapsulated docetaxel led to sustained release of the drug and BM localization. This experimental study showed that denosumab or nano-encapsulated docetaxel alone was associated with tumor relapse despite the initial antitumor and antimetastatic response. The combination therapy inhibited metastasis and tumor progression with minimal side effects such as bone loss. These findings suggest that improved chemotherapy with nanosystems and the RANK/RANKL pathway inhibitors can inhibit the interaction between tumor cells and the BME components and balance the activity of osteoblasts and osteoclasts (139).

**Table 2:** Novel therapeutic approaches for the treatment of bone-metastatic cancers.

Intervention	Mechanism of action and outcomes	Ref
**Denosumab + nano-encapsulated docetaxel**	• Inhibiting metastasis and tumor progression with minimal side effects such as bone loss • Inhibiting the RANK/RANKL pathway • Inhibiting the interaction between tumor cells and the BME components • Balancing the activity of osteoblasts and osteoclasts	(139)
**CPT-loaded pSiNP**	• Improving cytotoxic effect • Reducing orthotopic primary tumor growth • Prolonging survival rate • Inhibiting bone metastases	(140)
**ALN/FA-decorated PTX-loaded NPs**	• Alendronate has a high affinity for binding to bone tissue hydroxyapatite • Increasing paclitaxel toxicity through FA-TPGS binding • Reducing bone destruction and bone loss in tumor-bearing mice • Inhibiting tumor growth and bone metastasis *in vivo* with limited adverse effects on normal tissues	(141)
**Oxa (IV)@ZnPc@M + anti-PD-L1**	• Engineered macrophages carrying nanomedicine containing photosensitizer and oxaliplatin prodrug • Chemo/immunotherapy • Inducing the polarization of macrophages to the M1 phenotype • Eliminate primary tumor cells through chemo-photodynamic therapy and induction of immunogenic cell death • The combination of anti-PD-L1 with Oxa(IV)@ZnPc@M lead to the elimination of bone-metastatic tumor cells • Promoting tumor-specific immune response • Prolonging overall survival with minimum systemic toxicity	(142)
**CD204^+^IL-4R^+^ Macrophage ablation**	• Repressing bone metastasis development • Ablation of the IL-4R and CCR2 could remarkably suppress bone metastasis progression • Prolonging survival rate	(143)
**CA/ALN@FcB**	• Induce ROS in bone metastases • Alendronate has a high affinity to bone, and cinnamaldehyde is a potent ROS generator • Cinnamaldehyde also increases the intracellular H_2_O_2_ levels to reduce hypoxia	(144)
**BSA-coated gold clusters**	• Inhibiting osteoclastogenesis and osteolysis-mediated inflammation *in vivo* • Inhibiting the migration, invasion, and colony formation of MDA-MB-231 breast cancer cells *in vitro* • Inhibiting both MDA-MB-231 activated and RANKL-induced osteoclast formation from BM-derived mononuclear cells *in vitro* • Suppressing the expression of osteolysis-related factors in MDA-MB-231 cells • Inhibiting NF-κB pathway activation in BM-derived mononuclear cells • Decreasing the osteolysis *in vivo*	(145)
**BTZ@ZnPc-ALN**	• Generating ROS to induce mitochondrial damage under irradiation • Increasing the cytosolic levels of Ca^2+^ and GRP78 protein expression to promote excessive ER stress • Hindering tumor cell proliferation • Increasing and directing the blood circulation into the affected bone tissue • Restoring metastatic lesions	(146)
**Olaparib**	• Dual inhibitors of PARP-1 and PARP-2 • Inducing breast cancer-mediated bone metastasis via PARP-2, but not PARP-1, definitely in the myeloid lineage, but not in the tumor cells • Inducing differentiation of osteoclast and bone loss • Deletion of PARP2 in myeloid cells increases the frequency of immature myeloid cells in BM, impairing the expression of CCL3 by upregulating the β-catenin-mediated CCL3 transcriptional suppression • Impaired CCL3 expression by changing subpopulations of T cell led to the creation of an immunosuppressive environment	(147)
**LMWH-modified liposomes + alendronate + doxorubicin**	• LMWH is an antimetastatic agent and also enhances liposome blood circulation time • More effective doxorubicin delivery • Inhibiting tumor growth and metastasis	(148)
**Calcilytics**	• Inhibiting tumorigenesis effects of Ca^2+^-sensing receptor • Reducing bone metastasis	(149)
**Targeting exosome-derived miR-21**	• In patients with bone-metastatic breast cancer, serum exosomes levels of miR-21 were significantly increased • miR-21 derived from SCP28 cell exosomes regulates protein levels of the PDCD4 to induce osteoclastogenesis • Targeting miR-21 may be a potential therapeutic target for clinical diagnosis and treatment of bone-metastatic breast cancer	(150)
**AMD3100**	• Inhibiting acetylated KLF5-induced CXCR4 causes osteoclastogenesis and the formation of bone-metastatic lesions	(151)
**Radium-223 and anti-PDL-1**	• Radium-223 can prolong the survival rate in a part of patients with bone-metastatic prostate cancer • Radium-223 alters the DNA damage repair and bone-associated pathways • Changing the pattern of plasma-derived exosomes • Treatment of Myc-CaP mice models with a combination of anti-PDL-1 and Radium-223 inhibited exosome-derived PDL-1	(152)
**Radium-223 and Sipuleucel-T**	• In patients with bone-predominant, minimally symptomatic metastatic castration-resistant prostate cancer as compared with those who received combination treatment, participants who participated in the control arm experienced a 3.2-fold increase in T cell responses (based on proliferation index) • Patients in the combination arm were more likely to have a PSA decline of more than 50% and to demonstrate longer progression-free survival and overall survival	(153)
**PB@LC/D/siR**	• SREBP1 is an abnormal lipid metabolism regulator and could be involved in the metastasis and progression of tumor cells in bone metastatic prostate cancer • Combining the siRNA interferes with SREBP1 with docetaxel in a nano delivery system (PB@LC/D/siR) could inhibit tumor cells proliferation, migration, and invasion with high safety, deep tumor penetration, and decent bone protection at the tumor site • Reducing the expression of SREBP1 and stearoyl-CoA desaturase-1 (SCD1)	(154)
**GPRC5A knockout with CRISPR/Cas9**	• High expression of GPRC5A is associated with increased bone-metastatic lesions as well as a lower survival rate in patients with prostate cancer • Reducing cell proliferation via induction of cell cycle arrest at the G2/M phase in GPRC5A KO PC3 cells • Repressing bone metastasis in xenograft mice models	(155)
**Inhibition of S1P/S1PR**	• Ligation of S1P to S1PR initiates downstream signals involved in cell proliferation, differentiation, migration, and apoptosis • Additionally, S1P is considered a biological bridge between bone formation and bone resorption	(156)
**IPA-3**	• PAKs play a role in cancer and could be a potential target for cancer therapy • Inhibiting the tumor cell (RM1) proliferation and locomotion • Reducing prostate cancer-mediated bone remodeling *in vivo*	(157)
**Cryoablation**	• Palliation of painful bone metastases • The mean pain score decreased by 2.61 points between baseline and week 8 • The participant’s quality of life improved, their opioid doses alleviated, and their functional status remained unchanged for six months	(158)
**177Lu-DOTA-IBA**	• Effectively control the progression of bone metastasis • Enhance patient survival, and improve their quality of life, especially in advanced cases	(159)
**MOF**	• Potential for enhancing immunotherapy in bone metastatic prostate cancer • The nano-regulator effectively targeted the tumor site, induced immunogenic cell death, and blocked the immunosuppressive effects of TGF-β	(160)
**DZ@CPH**	• Reducing the activation of osteoclasts, leading to the inhibition of bone resorption • Inhibiting the invasion of TNBC cells into bone tissue by regulating the expression of proteins involved in apoptosis and invasion • Hindering the growth and spread of TNBC cells within the BME • Increasing the ratio of M1-type macrophages to M2-type macrophages in the bone metastasis tissue	(161)
**JNK-IN-8**	• Effectively suppressed tumor growth and bone metastasis in MCF7-BM cells	(162)
**BM-Evs contained miR-3190**	• Enhancing their ability to metastasize by reducing the expression ALKBH5 • The decreased levels of ALKBH5 worsen the pro-metastatic characteristics of HCC by modulating gene expression through both N6-methyladenosine-dependent and -independent mechanisms • In mouse models treated with BM-EVs, liposomes loaded with antagomir-miR-3190 and targeting HCC cells successfully suppress the progression of HCC	(163)
**NTZ**	• Affecting the function of the modified KLF5 • Increasing the expression of MYBL2 • Binding to the KLF5 protein, while the modified KLF5 bound to the promoter region of MYBL2 to activate its transcription • Attenuating the binding of the modified KLF5 to the MYBL2 promoter	(164)
**CLALN + mild-PTT**	• Significant inhibition of tumor progression by impairing autophagy and reducing the expression of PD-L1 protein induced by mild-PTT • Overcoming thermal resistance and alleviating immunosuppression • Effectively reduced osteolysis, which was not achieved by using CLALN alone or mild-PTT alone	(165)
**SPON2 Silencing** **Inhibition of NF-κB**	• Significantly reduced bone metastasis in a mouse model of ADC • Reducing the expression of MMP2 and MMP9 in metastatic bone tissues • Suppressing bone metastasis • Inhibition of NF-κB using a specific inhibitor attenuated the migration and invasion induced by SPON2 in ADC cells	(166)
**Activating GPR84**	• Significantly inhibited the formation of osteoclasts in the TME • Preventing osteolysis, the destruction of bone tissue, during CRC-induced bone metastasis	(167)
**Olaparib + PARP inhibitors + radium-223**	• The most common adverse events related to the treatment were fatigue (92%) and anemia (58%) • A positive outcome was indicated by a 58% radiographic progression-free survival (rPFS) rate at the 6-month mark • Out of the nine patients whose HRR gene status was evaluated, one patient exhibited a BRCA2 alteration (with an rPFS of 11.8 months), and another patient had a CDK12 alteration (with an rPFS of 3.1 months)	(168)
**NIR-PIT + Panitumumab-IR700 conjugate**	• Exerting a therapeutic effect on the bone metastatic lesions in the mice • Repairing bone destruction caused by the metastases, leading to the restoration of bone cortex continuity, similar to the healing process	(169)
**ZA**	• There was no significant difference in the time it took for the first SRE to occur between the two groups (P = 0.715, HR = 1.18, 95% CI = 0.48, 2.9) • The rate of SREs after 12 months was 17.6% (95% CI = 8.4, 30.9%) in the 4wk-ZA group and 23.3% (95% CI = 11.8, 38.6%) in the 8wk-ZA group, with no significant difference observed • No significant differences were found between the two groups for any of the secondary endpoints, and these endpoints did not vary among different treatment modalities • An eight-week interval for ZA administration does not increase the risk of SREs in patients with bone metastasis from lung cancer	(170)
**FLASH**	• The observed side effects were mild and in line with those seen in standard radiotherapy • Despite some brief episodes of increased pain at specific treated sites, most patients reported experiencing pain relief and a substantial reduction in discomfort at those locations	(171) NCT04592887
**177Lu-DOTA-IBA**	• Exhibited rapid elimination from the bloodstream, minimal absorption by soft tissues, and excretion through urine, with a specific bone accumulation • Alleviated pain in patients within a few days of administration, offering enduring relief without any harmful side effects	(159)

Recent studies demonstrated that drug-delivery nanosystems could consider potent therapeutic agents to improve the effectiveness of therapy in bone-metastatic cancers (172). For instance, Camptothecin (CPT), a nonspecific anticancer drug with high cytotoxicity and low water solubility properties, was loaded to improve the therapeutic effects of CPT porous silicon nanoparticles (pSiNP) was constructed, and treatment of MDA-MB-231BO cells with this nanosystem confirmed its cytotoxic effect. Humanized tissue-engineered bone constructs provided a humanized BME for breast cancer bone metastases in female NOD-SCID IL2Rgnull (NSG) mice. Outcomes showed that CPT-loaded pSiNP treatment reduced orthotopic primary tumor growth, prolonged survival rate, and significantly inhibited bone metastases (140). This investigation indicated that pSiNP could be a practical approach for targeted drug delivery of chemotherapeutic agents with deprived pharmacokinetic profiles.

A nanosystem containing poly (lactic-*co*-glycolic acid) (PLGA) coated with alendronate-modified D-α-tocopheryl polyethylene glycol succinate (ALN-TPGS) and folic acid-conjugated TPGS (FA-TPGS) was constructed as a vehicle for paclitaxel to enhance antitumor drug delivery in 4T1 tumors. Due to the presence of alendronate, this platform has a high affinity for binding to bone tissue hydroxyapatite. Correspondingly, tumor cells’ increased folate receptor expression enhances this nanosystem’s efficiency by increasing paclitaxel toxicity through FA-TPGS binding. ALN/FA-decorated PTX-loaded NPs also condensed bone destruction and loss in tumor-bearing mice. Moreover, this platform inhibited tumor growth and bone metastasis *in vivo* with limited adverse effects on normal tissues (141).

(Oxa (IV)@ZnPc@M) are engineered macrophages carrying nanomedicine containing photosensitizer and oxaliplatin prodrug. This system is designed as near-infrared light-activated drug vectors aiming to improve the outcomes of bone-metastatic tumors photo/chemo/immunotherapy. Oxa (IV)@ZnPc@M induces the polarization of macrophages to the M1 phenotype. Additionally, activated drugs by near-infrared light can concurrently eliminate primary tumor cells through chemo-photodynamic therapy and induction of immunogenic cell death.

Combining anti-PD-L1 with Oxa(IV)@ZnPc@M eliminates bone-metastatic tumor cells, promotes tumor-specific immune response, and expands overall survival with minimum systemic toxicity (142). Recently, a dual-function bone-targeting polymer vesicle with strong SPECT/CT imaging capability and drug delivery efficiency was fabricated for real-time diagnosis and killing of tumor cells. This study reported that SPECT/CT dynamically traced the drug delivery in the bone tumor rabbit models. Moreover, after 11 days of treatment with this platform, tumor size was pointedly decreased via inducing apoptosis and necrosis of the tumor cells (173). Another report stated that macrophage ablation could significantly repress bone metastasis development. Participated macrophages in bone metastases are commonly CD204^+^IL-4R^+^ and derived from CCR2^+^Ly6C^+^ inflammatory monocytes. Therefore, ablation of the IL-4R and CCR2 could remarkably suppress bone metastasis progression and extend the survival rate (143).

CA/ALN@FcB is another nanosystem composed of versatile alendronate-functionalized and cinnamaldehyde-loaded nanoscale coordination polymer fabricated to induce reactive oxygen species (ROS) in bone metastases. In this platform, alendronate has a high affinity to bone, and cinnamaldehyde is a potent ROS generator. Cinnamaldehyde also increases intracellular H_2_O_2_ levels to reduce hypoxia (144). Previous studies demonstrated that gold clusters could inhibit osteoclastogenesis and osteolysis-mediated inflammation *in vivo*. A study investigated the effects of bovine serum albumin (BSA)-coated gold clusters on bone-metastatic breast cancer in both laboratory settings and animal models. The results demonstrated that the gold clusters had a dose-dependent inhibitory effect on the migration, invasion, and colony formation of MDA-MB-231 breast cancer cells *in vitro*. Furthermore, the gold clusters were found to suppress the formation of osteoclasts from bone marrow-derived mononuclear cells *in vitro*, both when activated by MDA-MB-231 cells and when induced by RANKL. In addition, the gold clusters appeared to reduce the expression of factors related to osteolysis in MDA-MB-231 cells, consequently inhibiting the activation of the NF-κB pathway in bone marrow-derived mononuclear cells. Moreover, in animal experiments, a suggested dosage of 10 mg Au/kg.bw (body weight) of the gold clusters showed a noteworthy decrease in osteolysis *in vivo*. These findings indicate the potential of BSA-coated gold clusters as a therapeutic approach for managing bone-metastatic breast cancer by targeting both cancer cells and bone-related processes (145).

In a chemo-photodynamic therapeutic approach, ALN-functionalized bone-seeking nanoagent (BTZ@ZnPc-ALN) was fabricated to codelivery the photosensitizer Zinc phthalocyanine (ZnPc), and the bortezomib (BTZ) (a proteasome inhibitor) in bone metastases. Findings showed that BTZ@ZnPc-ALN could generate ROS to induce mitochondrial damage under irradiation. This nanosystem also elevated the cytosolic Ca2+ and GRP78 protein expression levels to promote excessive endoplasmic reticulum (ER) stress, hindering tumor cell proliferation in a synergetic manner. Another benefit of this therapeutic approach is increasing and directing blood circulation into the affected bone tissue, which can help restore metastatic lesions (146).

Olaparib is a United States Food and Drug Administration (FDA)-approved dual inhibitor of poly [ADP-ribose] polymerase 1 (PARP-1) and PARP-2 for the treatment of advanced ovarian and breast cancers. A study reported that Olaparib induced breast cancer-mediated bone metastasis via PARP-2, but not PARP-1, in the myeloid lineage, not in the tumor cells. Furthermore, deleting PARP-1 and PARP-2 or administering Olaparib could induce osteoclast differentiation and bone loss. Fascinatingly, the deletion of PARP2 in myeloid cells increases the frequency of immature myeloid cells in BM, impairing the expression of CCL3 by upregulating the β-catenin-mediated CCL3 transcriptional suppression. Impaired CCL3 expression by changing subpopulations of T cells leads to the creation of an immunosuppressive environment (147). These findings show that combination therapy with β-catenin inhibitors, CCL3, anti-RANKL or bisphosphonates, and PARP inhibitors can help treat bone-metastatic breast cancer.

Combining the low molecular weight heparin (LMWH) modified liposomes and alendronate as an anti-osteoporosis used more effective doxorubicin (an anticancer drug) delivery. LMWH is an antimetastatic agent that enhances liposome blood circulation time in this platform. Therefore, this system could significantly inhibit tumor growth and metastasis (148). The Ca^2+^-sensing receptor is a class-C G protein-coupled receptor (GPCR) involved in calciotropic processes via regulating the secretion of parathyroid hormone to preserve systemic calcium homeostasis. In addition, the Ca^2+^-sensing receptor can play a dual role in tumorigenesis, meaning it can be both a tumor suppressor and an oncoprotein. In breast cancer, the Ca^2+^-sensing receptor induces tumorigenesis and bone metastasis, while maternal breast tissue increases lactation. Due to the critical role of the Ca^2+^-sensing receptor, using its antagonists, such as calcilytics, can be a novel and effective therapeutic intervention for treating bone metastases caused by breast cancer. However, further studies are required in this field (149).

According to the available knowledge, exosomes as communication messengers could be involved in forming a pre-metastatic niche. An investigation reported that SCP28 cells-secreted exosomes promote osteoclast differentiation and activation, inducing bone lesion formation to restructure BME. In patients with bone-metastatic breast cancer, serum exosome levels miR-21 were significantly increased. It has been revealed that miR-21 derived from SCP28 cell exosomes regulates protein levels of the programmed cell death 4 (PDCD4) to induce osteoclastogenesis. These data designated that targeting miR-21 may be a potential therapeutic target for clinical diagnosis and treatment of bone-metastatic breast cancer (150). Bone-borne TGF-β can induce the transcription factor KLF5 acetylation in advanced prostate cancer-mediated bone metastases. Acetylated KLF5, by activating CXCR4, causes osteoclastogenesis and the formation of metastatic bone lesions. Following the upregulation of CXCR4 and its downstream signals, the production of IL-11 increased, activating the serum sonic hedgehog (Shh)/IL-6 paracrine signaling pathway. In addition, acetylated KLF5 is involved in docetaxel resistance mechanisms in bone-metastatic cancers, and CXCR4 antagonists such as AMD3100 could reverse these adverse effects (151).

Evidence demonstrated that Radium-223 could prolong the survival rate in some patients with bone-metastatic prostate cancer. A study showed that following treatment of mice with Radium-223, a significant alteration occurred in DNA damage repair and bone-associated pathways. Moreover, in patients with prostate cancer under treatment with Radium-223, the pattern of plasma-derived exosomes was changed, and exosome-derived PD-L1 was detected, which is associated with a low survival rate. To overcome this challenge, Myc-CaP mice models were treated with a combination of anti-PDL-1 and Radium-223, and the results were more promising than monotherapy with Radium-223 (152). On the other hand, it has been revealed that anti-PDL-1 antibody (Pembrolizumab) monotherapy inhibited tumors with a satisfactory safety profile in a subset of patients with bone-metastatic prostate cancer who were earlier treated with targeted endocrine therapy and docetaxel. Induced antitumor responses were durable and prolonged overall survival in patients with metastatic prostate cancer (174). Therefore, this type of combination therapy may increase the effectiveness of cancer therapy in patients with bone-metastatic prostate cancer.

In men with bone-predominant, minimally symptomatic metastatic castration-resistant prostate cancer, radium-223 was analyzed for the possibility of increasing peripheral immune responses to Sipuleucel-T as an autologous cellular immunotherapy. Compared with those who received combination treatment, participants in the control arm experienced a 3.2-fold increase in T cell responses (based on proliferation index). It was demonstrated that patients in the combination arm were more likely to have a more than 50% PSA decline and to show more prolonged progression-free and overall survival. Accordingly, the combination of Sipuleucel-T and radium-223, despite paradoxically lower immune responses observed, may increase clinical activity in men with asymptomatic bone mCRPC (153).

Sterol regulatory element-binding protein 1 (SREBP1), an abnormal lipid metabolism regulator, could be involved in metastasis and progression of tumor cells in bone-metastatic prostate cancer. It has been reported that combining the siRNA interferes SREBP1 with docetaxel in a nano delivery system (PB@LC/D/siR) could inhibit tumor cells proliferation, migration, and invasion with high safety, deep tumor penetration, and decent bone protection at the tumor site. Moreover, PB@LC/D/siR significantly reduced the expression of SREBP1 and stearoyl-CoA desaturase-1 (SCD1) (154).

High expression of GPRC5A is associated with increased bone-metastatic lesions and lower survival rates in patients with prostate cancer. A study on PC3 prostate cancer cells demonstrated that following GPRC5A knockout with CRISPR/Cas9, cell proliferation was significantly decreased via induction of cell cycle arrest at the G2/M phase in GPRC5A KO PC3 cells. Moreover, bone metastasis repressed GPRC5A KO PC3 cells in xenograft mice models (155). Sphingosine-1-phosphate (S1P) and its receptor S1PR expressed by osteoblasts and osteoclasts are other attractive therapeutic targets in bone-metastatic cancers because ligation of S1P to S1PR initiates downstream signals involved in cell proliferation, differentiation, migration, and apoptosis. Additionally, S1P is considered a biological bridge between bone formation and bone resorption (156). Evidence revealed that P21-activated kinases (PAKs) play a role in cancer and could be a potential target for cancer therapy. A study reported that targeting PAK1 kinase activity by an allosteric inhibitor (IPA-3) hindered the tumor cell (RM1) proliferation and locomotion. IPA-3 therapy also reduces prostate cancer-mediated bone remodeling *in vivo* (157).

An extreme cold treatment called cryoablation kills cancer cells with a thin needle (cryoprobe). The needle is inserted directly into the tumor and cooled with gas to destroy cancer cells (175). According to a clinical trial (NCT02511678) conducted on 66 patients receiving cryoablation, the mean pain score decreased by 2.61 points between baseline and week 8. Moreover, the study observed that participants who underwent cryoablation of metastatic bone tumors experienced significant improvements in their quality of life. Additionally, they required lower doses of opioids for pain management, indicating that cryoablation was effective in alleviating pain. Furthermore, the functional status of the participants remained stable over six months. These results demonstrate that cryoablation is a rapid and long-lasting pain relief option, leading to enhanced quality of life and presenting an alternative to opioid-based pain management for individuals with metastatic bone tumors (158).

A study aimed to investigate the fundamental characteristics of 177Lu-DOTA-IBA, a radiopharmaceutical comprehensively, and offer valuable guidance for its clinical utilization (159). The study’s findings revealed that 177Lu-DOTA-IBA possessed an impressively high radiochemical purity exceeding 98%. It exhibited favorable biological properties and demonstrated safety characteristics. The radiopharmaceutical displayed rapid clearance from the bloodstream, low uptake in soft tissues, and predominant excretion through the urinary system. Remarkably, it exhibited selective targeting and accumulation in bone tissues. Furthermore, the preliminary clinical translation study involving three patients who received treatment with 177Lu-DOTA-IBA reported significant and lasting pain relief within three days extending over two months. Importantly, no toxic side effects were observed.

Additionally, the study indicated that low doses of 177Lu-DOTA-IBA were effective and well-tolerated, devoid of significant adverse reactions (159). In conclusion, this study implies that 177Lu-DOTA-IBA holds excellent promise as a radiopharmaceutical for the targeted treatment of bone metastases. It has the potential to effectively control the progression of bone metastasis, enhance patient survival, and improve their quality of life, especially in advanced cases. Furthermore, its favorable pharmacokinetic characteristics and relatively straightforward preparation make it a strong candidate for future clinical applications.

A bone-targeted nano-delivery system, referred to as a nano-regulator, was developed to enhance immunotherapy in this specific context. The researchers assembled the nano-regulator using phytic acid (PA) and Fe3^+^ to create a nano-sized metal-organic framework (MOF) (160). They then encapsulated mitoxantrone (MTO), a chemotherapy drug, within this framework. At the cellular level, the nano-regulator demonstrated selective cytotoxicity towards RM-1 Prostat cancer cells while sparing immune cells. It also induces immunogenic cell death (ICD) in the tumor cells, which enhances their immunogenicity.

In addition, the nano-regulator was found to trigger ubiquitination of the TGF-β receptor (TGF-βR) on immune cells, subsequently leading to the receptor’s degradation. This unique mechanism of action acted as a nano-regulator, effectively inhibiting the functions of TGF-β, a cytokine known for its immunosuppressive effects within the TME. By blocking TGF-β signaling, the nano-regulator aimed to reverse the immunosuppressive effects and restore immune sensitivity in bone metastatic tumors, potentially enhancing the body’s ability to combat cancer cells in that specific setting. In animal studies, the researchers found that when administered intravenously, the nano-regulator exhibited prolonged blood circulation and selectively accumulated in bone metastatic sites. When combined with αCTLA-4, the nanoparticle demonstrated a robust anti-tumor effect.

Additionally, the treatment significantly alleviated bone destruction, reducing skeletal-related events associated with bone metastasis (160). This study provides a biocompatible nanomedicine approach that shows potential for enhancing immunotherapy in bone metastatic prostate cancer. The nano-regulator effectively targeted the tumor site, induced immunogenic cell death, and blocked the immunosuppressive effects of TGF-β (176). These findings represent a step towards restoring immune sensitivity in bone metastatic tumors and improving the effectiveness of immunotherapy in this challenging setting.

In a study, researchers developed calcium phosphate hybrid micelles loaded with docetaxel and zoledronate drugs, DZ@CPH (161). They aimed to create a therapeutic strategy that addresses the activation of osteoclasts (cells involved in bone resorption) and the invasion of triple-negative breast cancer (TNBC) cells into bone tissue. DZ@CPH demonstrated several beneficial effects. Firstly, it reduced the activation of osteoclasts, leading to the inhibition of bone resorption. Additionally, it inhibited the invasion of TNBC cells into bone tissue by regulating the expression of proteins involved in apoptosis and invasion. This suggests that DZ@CPH may hinder the growth and spread of TNBC cells within the bone microenvironment.

Moreover, DZ@CPH was found to increase the ratio of M1-type macrophages to M2-type macrophages in the bone metastasis tissue. This shift towards M1 macrophages, which possess anti-tumor properties, indicates that DZ@CPH may modulate the immune response in the BME and potentially enhance the anti-tumor immune response. Overall, DZ@CPH was demonstrated to disrupt the destructive cycle between bone metastasis growth and bone resorption. Targeting both aspects improved the therapeutic effectiveness in treating drug-resistant TNBC bone metastasis (161). This study provides valuable insights into a potential treatment approach that may have significant implications for addressing the challenges associated with bone metastasis in TNBC.

A study found that the protein c-Jun levels increased in MCF7-BM cells compared to the original cells (162). Additionally, the absence of c-Jun was observed to suppress tumor cell migration, transformation, and the ability to cause bone destruction (osteolysis). *In vivo*, experiments using a dominant-negative form of c-Jun resulted in smaller bone metastatic lesions and a lower occurrence of metastasis. Examination of bone metastatic lesions revealed varying expression of c-Jun. Moreover, overexpression of c-Jun in MCF7-BM cells established a detrimental cycle between these cells and osteoclasts, promoting migration induced by calcium and the release of BMP5, an osteoclast activator. The study presented compelling evidence that pharmacologically inhibiting c-Jun using a Jun amino-terminal kinase (JNK) inhibitor called JNK-IN-8 effectively suppressed tumor growth and bone metastasis in MCF7-BM cells.

Additionally, the downstream signals of c-Jun were found to be closely associated with the clinical prognosis of patients with the luminal subtype of breast cancer. This highlights the significance of c-Jun as a potential therapeutic target to prevent bone metastasis, specifically in luminal breast cancer cases. The expression of c-Jun was identified as a critical factor in promoting bone metastasis by creating a detrimental cycle within the bone microenvironment. These insights open up promising avenues for the development of subtype-specific therapies that can effectively tackle bone metastasis in luminal breast cancer. By targeting c-Jun, researchers and clinicians may have the opportunity to provide more tailored and effective treatments for patients with this particular subtype of breast cancer, ultimately leading to improved outcomes and better quality of life (162).

Extracellular vesicles (EVs) play a significant role in developing and spreading HCC and its metastasis (177). This study investigated the communication between primary HCC and bone lesions through Evs. However, understanding this process is currently limited, and the impact of bone metastasis on HCC progression has yet to be thoroughly explored. Researchers discovered that EVs derived from HCC cells that have metastasized to the bone (BM-EVs) localize to HCC cells at the primary tumor site and play a significant role in promoting the advancement of HCC. The underlying mechanism involves a specific microRNA called miR-3190-5p (miR-3190), which is found to be upregulated in both HCC cells present in bone lesions and the EVs derived from these cells. The miR-3190 present in BM-EVs is transferred to HCC cells at the primary site, enhancing their ability to metastasize. This is achieved by reducing the expression of a protein called AlkB homolog 5 (ALKBH5) in the recipient HCC cells. The decreased levels of ALKBH5 exacerbate pro-metastatic characteristics in HCC cells by modulating gene expression through both N6-methyladenosine-dependent and -independent mechanisms. These findings shed light on the role of EV-mediated communication between HCC cells in bone lesions and the primary tumor site. The upregulation of miR-3190 in BM-EVs and its subsequent transfer to HCC cells at the primary site significantly promote HCC metastasis. The involvement of ALKBH5 in this process highlights its potential as a therapeutic target for managing bone metastasis and halting the progression of HCC. Finally, in mouse models treated with BM-EVs, liposomes loaded with an antagonist of miR-3190 (antagomir-miR-3190) and targeting HCC cells successfully suppress the progression of HCC. The study findings demonstrate that BM-EVs derived from HCC cells in bone metastases trigger pro-metastatic events in primary HCC cells by transferring miR-3190, which targets ALKBH5. This highlights miR-3190 as a potential therapeutic target for inhibiting the progression of HCC in patients with bone metastasis (163).

TGF-β, abundant in bone tissue, is crucial in developing bone metastasis (127). However, directly targeting TGF-β or its receptors has proven to be complex. Previous studies have shown that TGF-β relies on the acetylation of a transcription factor called KLF5 at position K369 to regulate various biological processes, including cancer cell spread to the bone (127). This acetylated form of KLF5 (Ac-KLF5) and its downstream targets could be potential therapeutic targets for TGF-β-induced bone metastasis in prostate cancer (178). To identify agents that can suppress invasion, researchers conducted a spheroid invasion assay using prostate cancer cells expressing a modified version of KLF5, mimicking Ac-KLF5. Through screening 1987 FDA-approved drugs, nitazoxanide (NTZ), an anthelmintic agent, exhibited potent invasion inhibition in the assay. In a mouse model, NTZ significantly inhibited bone metastasis, both preventively and therapeutically (164). It also hindered the differentiation of osteoclasts, which are involved in bone metastasis induced by the modified KLF5. Further analysis revealed that NTZ binds to the KLF5 protein, while the modified KLF5 is bound to the promoter region of MYBL2, a gene known to promote bone metastasis in prostate cancer, to activate its transcription (164). NTZ was found to attenuate the binding of the modified KLF5 to the MYBL2 promoter. These findings suggest that NTZ could potentially be a therapeutic agent for bone metastasis induced by the TGF-β/Ac-KLF5 signaling pathway in prostate cancer and other types of cancer.

A novel therapeutic strategy involves utilizing liquid metal (LM) nanoparticles to address the limitations of mild photothermal therapy (mild-PTT) in treating deep and internal tumors. Mild-PTT is a less aggressive form of photothermal therapy, but it faces challenges such as thermal resistance, limited irradiation area, and penetration depth when targeting deep tumors (165). Additionally, tumor colonization in distant bone tissue leads to bone resorption, exacerbating tumor progression. To overcome these issues, researchers developed a novel approach using LM nanoparticles to enhance the effectiveness of mild PTT through autophagy activation. They loaded the LM nanoparticles and an autophagy activator called Curcumin (Cur) into zeolitic imidazolate framework-8 (ZIF-8), which was further functionalized with hyaluronic acid/alendronate (CLALN). This CLALN formulation offered several advantages, including good photothermal performance, drug release in acidic environments, and specific recognition and aggregation at bone metastasis sites. The combination of CLALN and mild PTT demonstrated significant inhibition of tumor progression by impairing autophagy and reducing the expression of PD-L1 protein induced by mild PTT. This combination therapy helped overcome thermal resistance and alleviate immunosuppression. Moreover, the combination effectively reduced osteolysis, which was not achieved using CLALN alone or mild PTT alone. The experimental results from this study indicate that the multifunctional LM-based nanoparticle combined with autophagy activation shows promise as a potential therapeutic strategy for effectively treating bone metastasis. This approach addresses the challenges of mild PTT in deep tumors and bone metastases and offers a promising solution for improving treatment outcomes in such cases (165).

Bone metastasis is a severe complication of lung adenocarcinoma (ADC) and significantly impacts patients’ survival and quality of life (179). Unfortunately, there is currently a lack of effective biomarkers for this incurable illness (180). While spondin-2 (SPON2) has been linked to metastasis and cancer advancement, its specific role in bone metastasis within lung ADC patients remains poorly comprehended (181, 182). To shed light on the matter, a study was undertaken to investigate the impact of SPON2 on bone metastasis in ADC (166). In laboratory experiments, the researchers discovered that elevating SPON2 in ADC cells resulted in increased migration, invasion, and epithelial-to-mesenchymal transition.

On the contrary, inhibiting SPON2 suppressed these processes, underscoring its significant influence on the metastatic behavior of ADC cells. These results were further supported by a mouse model of ADC, where silencing SPON2 substantially decreased bone metastasis. The underlying mechanism revealed that SPON2 activated the nuclear factor-κB (NF-κB) signaling pathway, leading to the upregulation of matrix metalloproteinase 2 (MMP2) and MMP9, both known to promote cancer cell migration and invasion. Blocking NF-κB with a specific inhibitor mitigated the migration and invasion induced by SPON2 in ADC cells.

Moreover, the study observed higher SPON2 expression in metastatic bone tissues than primary ADC tissues. This upregulation of SPON2 was positively correlated with increased levels of MMP2 and MMP9 in metastatic bone tissues, further supporting its role in promoting bone metastasis (166). These findings underscore the critical involvement of SPON2 in ADC, as it triggers the NF-κB pathway, ultimately promoting bone metastasis. Therefore, targeting SPON2 in future drug development may hold promise in preventing or treating bone metastasis in ADC patients.

The role of GPR84, a receptor found in bone marrow-derived monocytes/macrophages (BMMs), in bone metastasis of CRC was investigated, and the findings revealed that the expression of GPR84 in BMMs was progressively downregulated during CRC-induced bone metastasis (167). Activating GPR84 significantly inhibited the formation of osteoclasts in the TME. The MAPK pathway was identified as the mediator of GPR84 in osteoclast formation. Furthermore, the study found that interleukin-11 (IL-11) played a role in inhibiting the expression of GPR84 in the TME by inactivating STAT1. Additionally, activating GPR84 helped prevent osteolysis, the destruction of bone tissue, during CRC-induced bone metastasis (167). Based on these results, it was concluded that CRC cells downregulate GPR84 expression in BMMs to promote osteoclastogenesis, and IL-11 is involved in this process by inhibiting GPR84 expression through STAT1 inactivation. Therefore, GPR84 can be a therapeutic target for attenuating bone destruction caused by CRC metastasis.

A multicenter phase I dose escalation study was conducted to assess the safety and effectiveness of combining olaparib, a drug that inhibits DNA repair mechanisms known as PARP inhibitors, with radium-223, a radiopharmaceutical that causes DNA damage, in patients with metastatic castration-resistant prostate cancer (mCRPC) (168). The study involved multiple centers and focused on escalating the dosage of the two drugs. The study observed specific toxicities that could limit the dosage, including cytopenias, fatigue, and nausea. However, no such toxicities were observed during the observation period. Instead, delayed toxicities were considered when determining the recommended phase II dosage (RP2D). The RP2D for the combination of olaparib and radium-223 was established as 200 mg taken orally twice daily. The most common adverse events related to the treatment were fatigue (92%) and anemia (58%). A 58% radiographic progression-free survival (rPFS) rate indicated a positive outcome at the 6-month. Out of the nine patients whose HRR gene status was evaluated, one patient exhibited a BRCA2 alteration (with an rPFS of 11.8 months), and another had a CDK12 alteration (with an rPFS of 3.1 months). Based on these findings, it was determined that olaparib could be safely combined with radium-223 at the recommended dosage of 200 mg taken orally twice daily. Early clinical benefits were observed, and further investigation will be carried out in a phase II study to assess the potential effectiveness of this combination therapy (168).

A promising and innovative technique known as near-infrared photoimmunotherapy (NIR-PIT) shows great potential in treating bone metastases. NIR-PIT involves using antibodies labeled with a substance called IRDye700DX (IR700), which becomes activated when exposed to a near-infrared light (183). To explore the effectiveness of NIR-PIT in addressing bone metastases, a study was conducted using a mouse model implanted with bone metastases. This model was created by injecting a human triple-negative breast cancer cell line, MDAMB468-GFP/luc, into the caudal artery of the mice. The researchers then applied NIR-PIT by using an anti-EGFR antibody named panitumumab-IR700 conjugate to treat the bone metastatic lesions in the mice. Through bioluminescence imaging and histological assessment, the study found that EGFR-targeted NIR-PIT demonstrated a therapeutic effect on the bone metastatic lesions in the mice. Furthermore, micro-CT scans revealed that repeated application of NIR-PIT led to the repair of bone destruction caused by metastases. This repair process resulted in the restoration of bone cortex continuity, akin to the natural healing process. These findings strongly suggest that NIR-PIT holds significant promise as a potential clinical treatment for bone metastases. By utilizing near-infrared light and targeted antibodies, this approach shows the ability to address bone metastases and facilitate bone tissue repair effectively. As a result, NIR-PIT could become a viable treatment option for patients with bone metastases (169).

A randomized phase 2 trial was conducted at multiple Japanese hospitals to evaluate the feasibility of administering Zoledronic acid (ZA) to patients with lung cancer and bone metastases either every four weeks (4wk-ZA) or every eight weeks (8wk-ZA) (170). The primary objective was to assess if there was any significant difference in the time it took for the first skeletal-related event (SRE) to occur between the two groups. The results indicated that there was no significant difference in the occurrence of the first SRE between the 4wk-ZA group and the 8wk-ZA group (P = 0.715, Hazard Ratio [HR] = 1.18, 95% Confidence Interval [CI] = 0.48, 2.9). Additionally, the study examined the rate of SREs after 12 months and found it to be 17.6% (95% CI = 8.4, 30.9%) in the 4wk-ZA group and 23.3% (95% CI = 11.8, 38.6%) in the 8wk-ZA group, with no significant difference observed between the two groups. Furthermore, the trial investigated several secondary endpoints, but no significant differences were observed between the two treatment groups. Importantly, the outcomes did not vary among different treatment modalities. Based on these findings, the study concludes that administering Zoledronic acid at an eight-week interval does not increase the risk of skeletal-related events in patients with bone metastasis from lung cancer. Therefore, an eight-week dosing interval for ZA could be considered a viable clinical option for these patients, potentially offering convenience and flexibility in their treatment regimen without compromising efficacy (170).

The FAST-01 study (NCT04592887) recently assessed the viability and safety of a pioneering technique in radiotherapy, known as FLASH therapy, specifically for patients with extremity bone metastases (171). In this trial, ten participants underwent palliative treatment using this innovative approach. The evaluation encompassed technical feasibility, treatment-related adverse effects, and the effectiveness of pain relief at the targeted sites. The findings indicated the attainability of FLASH therapy at an exceptionally high rate without encountering technical impediments or delays. On average, patients spent roughly 18.9 minutes during treatment, and the observed side effects were mild and in line with those seen in standard radiotherapy. Despite some brief episodes of increased pain at specific treated sites, most patients reported experiencing pain relief and a substantial reduction in discomfort at those locations. These results imply that ultra-high-dose-rate proton FLASH therapy is feasible and practical, demonstrating a safety profile comparable to traditional radiotherapy. Such promising outcomes encourage further investigation and potential adoption of FLASH therapy in cancer care, signifying a significant advancement in radiotherapy research and its practical use in clinical settings (171).

Another recent study centered on a new method to diagnose and treat bone metastases using a recently developed radiopharmaceutical called 177Lu-DOTA-IBA (159). This study explored the fundamental biological traits of this radiopharmaceutical and assessed its potential for practical use in clinical settings. By refining the labeling process and conducting tests in laboratory conditions, the 177Lu-DOTA-IBA displayed high purity concerning its radioactive components, favorable biological characteristics, and safety. It exhibited rapid elimination from the bloodstream, minimal absorption by soft tissues, and excretion through urine, with a specific bone accumulation. Imaging outcomes from experiments on mice and an initial human trial showed promising results. Notably, the treatment alleviated pain in patients within a few days of administration, offering enduring relief without any harmful side effects. This radiopharmaceutical is a viable and efficient choice for targeted treatment, potentially managing the progression of bone metastases and improving the lives of individuals with advanced stages of this condition. Its ease of preparation, favorable characteristics related to how the body processes it, and the absence of significant adverse reactions position 177Lu-DOTA-IBA as a hopeful prospect for enhancing patient outcomes in this challenging medical field (159).

## 7 Challenges in bone-metastatic cancer treatment

The treatment of bone-metastatic cancers poses significant challenges due to the complex nature of the disease and its interactions with the BME (184). While advancements have been made in cancer therapy, effectively targeting and eradicating tumor cells that have spread to the bones remains formidable. This section explores some of the key challenges encountered in treating bone-metastatic cancers.

Achieving a curative outcome becomes exceedingly difficult once cancer cells metastasize to the bone. The bone tissue provides a supportive environment for tumor cells to establish and grow (185). Conventional treatment modalities such as surgery, chemotherapy, and radiation therapy often fail to eradicate metastatic tumors from the bone, leading to a focus on palliative care and the management of symptoms (186). Metastatic tumor cells in the bone exhibit enhanced resistance to conventional cancer therapies (178, 187). The unique microenvironment of the bone, consisting of specialized cells and ECM components, contributes to the development of therapy resistance (188). This resistance can hamper the effectiveness of systemic treatments, rendering them less potent in controlling tumor growth and progression (189). Bone metastasis frequently leads to skeletal complications, including bone pain, fractures, spinal cord compression, and hypercalcemia (190). These complications significantly impact the quality of life for patients and require specialized management approaches (191). The treatment of bone metastasis must address the primary tumor and manage the skeletal-related events associated with the disease. Bone metastasis is characterized by the formation of multiple lesions within the skeletal system, and these lesions can display varying degrees of aggressiveness and sensitivity to treatment (192). The heterogeneity of metastatic lesions poses a challenge in delivering targeted therapies that can effectively eradicate all tumor sites while minimizing damage to healthy bone tissue (193).

Moreover, early detection and accurate diagnosis of bone metastasis remain challenging (194). Imaging techniques such as bone scans, X-rays, and CT scans are commonly used, but they may not detect small metastatic lesions or accurately assess the extent of the disease (195). Accordingly, improved diagnostic methods are needed to facilitate early intervention and personalized treatment strategies.

Overcoming these challenges requires a multidisciplinary approach combining innovative therapeutic strategies, imaging and diagnostic techniques advancements, and a comprehensive understanding of the BME (196). By addressing these challenges head-on, researchers and clinicians aim to improve outcomes for patients with bone-metastatic cancers, enhance the quality of life, and develop more effective treatment options tailored to the unique characteristics of this disease.

## 8 Concluding remarks

Treating human malignancies characterized by metastases to distant organs has long presented significant challenges in oncology. One particular group of cancers that pose formidable difficulties are those that metastasize to the bone, leading to a range of complications for patients, including excruciating pain, bone fractures, spinal cord compression, hypercalcemia, and dismal survival rates. Existing therapies for metastatic bone cancers have fallen short of providing a cure, offering partial inhibition of metastasis and tumor progression within the bone tissue. Consequently, the advent of innovative approaches employing nanosystems or immunotherapeutic methods holds immense promise in enhancing treatment efficacy by leveraging combination therapies or augmenting the delivery of anticancer agents. Nevertheless, it is imperative to underscore that further investigations are imperative to ascertain optimal combinations with minimal toxicity and side effects while maximizing therapeutic efficacy, particularly during human clinical trial phases. By embracing such endeavors, we can potentially revolutionize the landscape of metastatic bone cancer treatment, providing new hope for patients facing this formidable disease.

## Author contributions

HL: Writing – original draft. BW: Writing – original draft. KJ: Writing – review & editing. YC: Writing – review & editing.
